# Artificial Intelligence in Drug Discovery and Development: Raising Quality per Decision

**DOI:** 10.1055/a-2810-8972

**Published:** 2026-03-05

**Authors:** Shota Furukawa, Hiroyuki Uchida, Taishiro Kishimoto

**Affiliations:** 1 Department of Neuropsychiatry, Keio University School of Medicine, Tokyo, Japan.; 2 IQVIA Services Japan G.K., Tokyo, Japan; 3 Center for the Promotion of Interdiciplinary Research in Medicine and Life Science, Keio University School of Medicine and Graduate School of Medicine, Tokyo, Japan

**Keywords:** artificial Intelligence, drug discovery, drug design, drug repositioning, clinical trials as topic

## Abstract

Drug research and development continuously encounters prolonged timelines, escalating costs, and high attrition rates. In this narrative review, we integrated recent advances in artificial intelligence across target identification, drug repurposing, de novo molecular design, structural biology, safety prediction, and artificial intelligence-supported clinical development, aligning these innovations with evolving global regulatory frameworks. Predictive and interpretable artificial intelligence could enhance the quality of decision-making throughout the research and development process when combined with causal or mechanistic priors, synthesis-aware and physics-informed molecular design, external validation with clear applicability domains, and governance systems aligned with multiple regulatory guidelines and qualified digital endpoint applications. Case studies of artificial intelligence-assisted discovery and repurposing demonstrate shorter development timelines, improved compound quality, and higher-level early-phase success, while underscoring challenges such as overfitting, model generalizability, and dataset bias. Establishing a context-of-use-based “credibility plan” and adopting equity-by-design through the inclusion of non-European datasets and subgroup performance evaluation are essential for achieving generalizable impact. Artificial intelligence integration with new approach methodologies and adaptive or covariate-adjusted clinical trials may help reduce development inefficiency without compromising scientific or ethical rigor.

## Introduction: challenges and advances in drug research and development

Biopharmaceutical research and development (R&D) remains lengthy, costly, and inefficient, with a development period from discovery to approval typically requiring 10–15 years 1​ ​2​ and an estimated capitalized cost per approved drug of 1.1–2.6 billion USD (failed candidates included). 3​ Despite advances in translational science and trial design, the overall success rates remain low: among 12,728 phase transitions (2011–2020), only 7.9% of Phase I candidates achieved approval. 4​

Artificial intelligence (AI) and machine learning (ML) have progressed from proofs of concept to practical deployment across the biopharmaceutical pipeline. In discovery, data-driven target identification, structure-informed molecular design, and *in silico* developability profiling accelerate design-test cycles. In parallel with these advances in discovery and design, psychiatry has made major mechanistic progress. The U.S. Food and Drug Administration (FDA) approved xanomeline–trospium (KarXT; Cobenfy), the first schizophrenia therapy in more than seven decades to act through muscarinic acetylcholine receptor (M1/M4) modulation rather than dopamine receptor antagonism. 5​ ​6​ This approval illustrates renewed interest in cholinergic signaling as a therapeutic pathway. Concurrently, AI-based analyses of M1 receptor pharmacology are advancing rapidly, suggesting that future psychiatric drug discovery may increasingly benefit from computational and data-driven approaches. 7​

In development, AI supports patient selection, trial simulation, and the early detection of safety signals using real-world and digital health data. Recent reviews from academia and regulators emphasize the breadth of these applications. 8​ ​9​ The International Council for Harmonisation (ICH) of Technical Requirements for Pharmaceuticals for Human Use E6(R3) Good Clinical Practice (GCP) guideline establishes a proportionate, risk-based framework for digitalized trials, defining expectations for data integrity, governance, and lifecycle validation of computerized systems. 10​

Although not AI-specific, regulators are also piloting measures to accelerate review timelines. In October 2025, the FDA launched the Commissioner’s National Priority Voucher program, granting priority review for products addressing national needs. Early reports indicated markedly shorter review durations than those undertaking standard pathways. 11​ ​12​

**Fig. 1:**
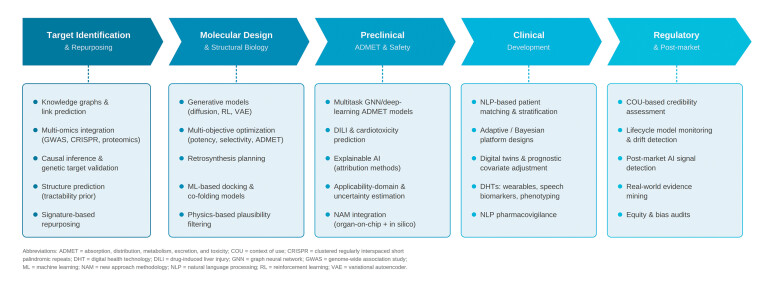
Overview of AI applications across the drug development pipeline.png. Each stage lists representative AI/ML methods discussed in this review. See Tables 1 2 3 4 for detailed descriptions of individual approaches, regulatory frameworks, and governance considerations. ADMET, absorption, distribution, metabolism, excretion, and toxicity; AI, artificial intelligence; COU, context-of-use; CRISPR, clustered regularly interspaced short palindromic repeats; DHT, digital health technology; DILI, drug-induced liver injury; GNN, graph neural network; GWAS, genome-wide association study; ML, machine learning; NAM, new approach methodology; NLP, natural language processing; RL, reinforcement learning; VAE, variational autoencoder.

In this review, we summarized recent advances in (i) target identification and repurposing, (ii) *de novo* molecular design and structural biology, (iii) preclinical absorption, distribution, metabolism, excretion, and toxicity (ADMET) and safety modeling, and (iv) AI-enabled clinical development, and (v) regulatory considerations and post-market surveillance ( Fig. 1 ), within their regulatory and ethical contexts. We argue that predictive, interpretable AI (aligned with risk-based GCP principles and externally validated) could lower attrition by improving the early candidate quality and shortening the drug lag through more efficient trials and reviews.

### Literature search and selection

This narrative review synthesizes literature identified through a combination of database searches (PubMed and Google Scholar), forward and backward citation tracking, and targeted retrieval of grey literature. We searched for publications using terms related to AI, ML, drug discovery, drug development, clinical trials, ADMET, and digital health, without imposing strict date restrictions, while emphasizing recent advances through late 2025. Given the rapid evolution of this field, we also incorporated regulatory guidance documents (FDA, European Medicines Agency [EMA], Pharmaceuticals and Medical Devices Agency [PMDA], and ICH), industry press releases, and technical reports from key organizations to capture emerging practices not yet fully reflected in the peer-reviewed literature. Case studies were selected to illustrate methodological diversity and translational progress, prioritizing examples with documented clinical advancement, regulatory interaction, or independent validation. This review does not follow formal systematic review protocols; rather, it aims to provide a comprehensive, clinically oriented overview of current AI applications across the drug development lifecycle.

## Artificial intelligence in target identification and drug repurposing

Selecting the right biological target remains critical to clinical success. Genetically validated targets are roughly twice as likely to reach approval, providing the empirical basis for modern AI frameworks to link genetic and causal data to systematic target prioritization and repurposing. 13​

## Novel target identification

Modern approaches integrate evidence from (i) human genetics (genome-wide association study, colocalization, and burden analyses), (ii) functional genomics via clustered regularly interspaced short palindromic repeat screens, (iii) transcriptomic and proteomic signatures, and (iv) structured or text-mined biomedical knowledge. Platforms such as Open Targets combine these sources into transparent target-disease scores, while DepMap quantifies gene essentiality across hundreds of cell lines for tractability. 14​ 15​ ​16​

AI-based hypothesis generation increasingly applies biomedical knowledge graphs (KGs), that is, networks, linking genes, proteins, diseases, and drugs through causal or functional edges. Foundational resources like Hetionet/Project Rephetio demonstrated that graph traversal and link-prediction could prioritize plausible target-disease pairs, inspiring probabilistic and semantics-enhanced KG–ML methods. By integrating data from genetics, pathways, and adverse-effect profiles, KGs allows for explainable and systematic therapeutic hypothesis ranking. 17​ 18​ ​19​

For targets without experimental structures, AlphaFold has revolutionized structure-based drug design by providing highly accurate predictions, with a public database currently containing over 200 million proteins. 20​ The 2024 AlphaFold 3 (AF3) model has been further extended to protein–ligand and nucleic acid complexes via a diffusion architecture, outperforming physics-based tools on the PoseBusters benchmark. 21​ In hepatocellular carcinoma, Insilico Medicine used PandaOmics and Chemistry42 to design a CDK20 inhibitor (Kd≈ 9 µM) within 30 days, demonstrating accelerated target-to-hit translation. 22​

Best practices emphasize confidence-aware use and cross-validation as follows. (i) Docking to predicted structures should use confidence- or template-guided refinement; naïve AF2 docking underperforms crystallographic baselines, although AF3 narrows this gap. 21​ 23​​ ​24​ (ii) KG-based hypotheses require stress-testing for leakage and bias: they detect symmetric or inverse edges, use leakage-robust splits (e.g., UMLS-43), adjust for degree confounding, and confirm links across literature, patents, and clinical data. 25​ 26​ ​27​ (iii) Bayesian colocalization analyses should distinguish shared from distinct causal variants (report PP3/PP4) and justify priors; joint-causality priors around 5×10 ^−6^ yield robust inference, with conditioning or masking for multiple signals. 28​ ​29​

### Artificial intelligence-driven drug repurposing

Drug repurposing exploits existing pharmacology and safety data to accelerate development at lower cost. Repurposed agents often reach the market within 3–12 years (faster and significantly cheaper than *de novo* programs) although success is indication- and strength of the evidence-dependent. AI now amplifies this efficiency by mapping drug–target–disease relationships across omics, electronic health records (EHRs), and literature using network- and graph-based ML and causal inference methods. 30​ ​31​

During the COVID-19 pandemic, Benevolent AI’s KG identified the Janus-kinase inhibitor baricitinib as a blocker of AAK1/GAK-mediated viral entry. Clinical trials confirmed benefit, leading to FDA Emergency Use Authorization in 2020 and illustrating how graph-driven repurposing can deliver validated therapies under compressed timelines. 32​ 33​ 34​ 35​ ​36​

COVID-era KGs such as CoV-KGE prioritized 41 drug–target–disease candidates, later validated by clinical trials and literature studies, demonstrating how graph embeddings and link prediction efficiently triage large candidate sets by integrating virus-host, pathway, and phenotypic information. 37​ ​38​

AI-generated repurposing hypotheses require tiered validation: (i) Mechanistic plausibility: Define a clear mechanism of action, including on- and off-target effects, and seek orthogonal support such as the reversal of disease signatures in connectivity map/LINCS profiles reproduced across cell types and assays. (ii) Bias control: Use prespecified negative controls, audit for data or label leakage (e.g., inverse or symmetric relationships in KGs), and test sensitivity to scoring, preprocessing, and hyperparameters. (iii) Experimental confirmation: Algorithmically ranked candidates must undergo confirmatory preclinical studies and prospectives and powered clinical trials. Because CMap/LINCS signatures are normalization-sensitive, replicate and triangulate across modalities (e.g., transcriptomics plus drug–gene–disease KGs). 39​ ​40​

These approaches are particularly relevant to psychiatry, where the genetic architecture of disorders such as schizophrenia and major depressive disorder is highly polygenic and the causal biology remains incompletely understood. AI-driven KGs and multi-omic integration offer a systematic means to prioritize novel targets beyond the monoamine hypothesis, as exemplified by the recent validation of muscarinic receptor modulation in schizophrenia.

## Artificial intelligence-driven molecular design and structural biology

Generative AI now spans a simplified molecular input line entry system (SMILES) and self-referencing embedded string (SELFIES)-based sequence models, graph generators, and 3D-equivariant diffusion systems, allowing for *de novo* design under multiple objectives such as potency, selectivity, ADMET, and synthetic accessibility. Meanwhile, structure prediction has evolved from single-chain to interaction-aware models, strengthening structure-based design. Taken together, these advances shorten design-make-test-analyze cycles and enhance candidate quality when paired with rigorous external validation, synthesis checks, and physics-based constraints. 41​ ​42​

### Generative artificial intelligence for De Novo molecular design

Modern generative pipelines integrate complementary model families: (i) sequence models (RNNs, Transformers on SMILES/SELFIES) for rapid scaffold exploration, (ii) graph-based generators for topology-aware control, and (iii) 3D-equivariant diffusion models (EDMs) producing atomistic conformations that preserve geometry and electrostatics. Multi-objective optimization (via reinforcement learning (RL) or constrained sampling) balances potency, selectivity, and developability, with open-source tools such as REINVENT 4 allowing for prospective molecule generation. 43​ 44​ ​45​

Generative tensorial RL produced DDR1 kinase inhibitors within 21 days under explicit synthesizability constraints. 46​ Clinically, the DSP-1181 program (Exscientia×Sumitomo Dainippon) advanced a 5-HT1A agonist to Phase I in 2020 after<12 months of exploratory research and≈350 synthesized compounds, compared with typical central nervous system (CNS) timelines of 4.5–5 years and thousands of compounds. 47​ ​48​

Models such as E(3)-EDM and GeoDiff generate 3D structures directly. At the same time, conditional diffusion enables (i) pose generation (DiffDock) with higher PDBBind accuracy and (ii) pocket-conditioned ligand design (DiffSBDD). These models encode shape complementarity yet still face residual physical-plausibility limits. 44​ 49​​ 50​ ​51​

Synthetic-feasibility filters (e.g., synthetic accessibility score) and computer-aided synthesis planning tools such as AiZynthFinder and ASKCOS prune unsynthesizable candidates and rank routes by cost or risk, while robotic and flow-chemistry systems close the design-make-test loop. 52​ 53​ ​54​

Common pitfalls include reward hacking, mode collapse, and missed activity cliffs. Mitigations involve multi-objective (Pareto) scoring, applicability-domain and out-of-distribution checks (OECD quantitative structure–activity relationship [QSAR] principles), diversity-aware RL with replay buffers or regularization, and prospective assays with negative controls, supported by evidence from RL and QSAR benchmarks. 55​ 56​ 57​ 58​ 59​ 60​ 61​ ​62​

### Protein structure prediction and structure-based drug design

Reframing molecular docking as a generative diffusion task (e.g., DiffDock) improves near-native pose accuracy on benchmarks such as PDBBind and maintains performance for computationally folded structures, where classical methods often degrade. Yet, PoseBusters demonstrated that various ML-based docking outputs remain chemically implausible without post-filtering by physics- or cheminformatics-based checks. When physical validity is enforced, classical methods could still outperform deep-learning approaches. This field is thus converging on hybrid strategies that pair ML-generated poses with physics-based refinement and PoseBusters-style plausibility filters. 49​ ​63​

Beyond docking and complex-aware predictors like AlphaFold 3, proprietary co-folding models directly predict 3D protein–ligand complexes from sequences and ligand structures. DragonFold (Charm Therapeutics) reportedly achieves the state-of-the-art pose accuracy and integrates with free-energy perturbation workflows for affinity estimation, with programs advancing toward the clinic. These models capture induced-fit effects and reduce setup burden; yet, independent studies warn of overfitting and violations of interaction physics without orthogonal validation. Accordingly, co-folded poses should be treated as hypotheses, subject to PoseBusters-style plausibility checks, mutagenesis or biophysical assays, and prospective validation before decision-grade use. 64​ 65​ 66​ ​67​

Multiple studies have cautioned that out-of-the-box docking to AF2 models can underperform when compared with docking to crystal structures in virtual screening. Performance improves with confidence-aware model selection, binding-site refinement, and post-docking minimization. 68​ ​69​

For CNS drug discovery, generative design faces additional constraints including blood–brain barrier penetration, off-target receptor selectivity across closely related aminergic and cholinergic receptors, and the need for favorable metabolic profiles to avoid CYP-mediated drug–drug interactions common in psychiatric polypharmacy. These requirements make multi-objective optimization and synthesis-aware design especially critical in the psychiatric context.

## Preclinical optimization and safety assessment (absorption, distribution, metabolism, excretion, and toxicity)

Safety and developability remain major causes of attrition. A cross-company analysis of four large pharmaceutical companies identified non-clinical toxicology as the leading cause of failure (~40%), with human pharmacokinetics and safety often emerging as late-stage liabilities, underscoring the need for earlier, model-based triage. 70​ Drug-induced liver injury (DILI) is a persistent contributor to clinical failure and post-approval withdrawal, making it a key end point for in-silico screening and microphysiological validation. 71​ ​72​

### Integrated absorption, distribution, metabolism, excretion, and toxicity modeling and safety assessment

Integrated computational modeling across ADMET domains now underpins preclinical decision-making and early risk triage. Recent advances have expanded the predictive scope from single-end point QSARs to multitask graph-based and deep-learning frameworks capable of modeling interconnected pharmacokinetic and toxicological processes. Alongside these advances, explainability and methodological guardrails have become central for regulatory acceptance, ensuring that AI-based predictions remain transparent, chemically valid, and mechanistically interpretable. Table 1 summarizes representative tools and model families across these domains, with corresponding references cited within the table. 73​ 74​ 75​ 76​ 77​ 78​ 79​ 80​ 81​ 82​ 83​ 84​ 85​ 86​ 87​ 88​ ​89​

**Table:** **Table 1** Summary of integrated ADMET modeling and safety assessment approaches

Scope/focus	Representative tools/models	Key features and modeling approaches	Validation and performance	Limitations/notes	References
Foundational tools	pkCSM; ADMETlab 2.0	Graph-based molecular signatures predicting multiple ADMET properties; ADMETlab 2.0 covers>80 physicochemical, PK, and toxicity end points (including transporter- and DDI-related excretion risks)	Widely used for early developability screening	Baseline models	73​ ​74​
Distribution (BBB and CNS exposure)	ML models for BBB permeability	Feature sets include polarity descriptors and dynamic PSA	Improved evaluation standards for generalization	Meta‑analyses propose improved evaluation standards for generalization; emphasize robust external validation	75​ ​76​
Metabolism and DDIs (CYP450 liabilities)	Multitask GNNs; hybrid ML+docking pipelines	Predicts CYP450 inhibition and substrate status with multitask learning and structure-based features	Strong internal AUCs; ML-augmented docking improves transfer across chemical space	Out-of-domain performance remains limited	77​ 78​ ​79​
Data-driven toxicology	DeepTox (Tox21); HelixADMET; ADMET-AI	Deep learning frameworks with multi-task training and self‑supervised pretraining with multi‑stage transfer	HelixADMET improves accuracy by≈4% over earlier systems, particularly when labeled data are scarce; ADMET‑AI achieves high AUROCs across toxicity and DILI end points	Data imbalance and label scarcity still challenging	80​ 81​ ​82​
Clinical signal and interpretability	Phase I AI-designed molecules (≈80–90% success, *n* ≈ 24); SHAP, LIME, SME explainers	Attribution of ADMET or toxicity predictions to interpretable molecular substructures	Facilitates mechanistic documentation and transparent safety reviews	Early success data are limited and preliminary	83​ 84​ ​85​
Methodological guardrails	Rigorous data curation; scaffold- or time-aware splits; model calibration; synthetic feasibility checks (SELFIES, RAscore, AiZynthFinder)	Combines bias control and reward-hacking mitigation with explainable AI (XAI) methods (SHAP, LIME, and SME)	Enhances model robustness and chemical plausibility	Hallucinations and non-viable synthetic routes remain risks in generative AI	84​ 86​​ 87​ 88​ ​89​

Abbreviations: ADMET, absorption, distribution, metabolism, excretion, and toxicity; AI, artificial intelligence; AUC, area under the curve; AUROC, area under the receiver operating characteristic curve; BBB, blood–brain barrier; CNS, central nervous system; CYP450, cytochrome P450; DDI, drug–drug interaction; DILI, drug-induced liver injury; GNN, graph neural network; LIME, local interpretable model-agnostic explanations; ML, machine learning; PK, pharmacokinetic(s); PSA, polar surface area; SELFIES, self-referencing embedded strings; SHAP, Shapley additive explanations; SME, subgraph mask explanation; XAI, explainable AI.

Note: An overview of representative computational tools and model families used for integrated ADMET prediction and safety assessment. The table summarizes foundational frameworks, domain-specific models for distribution and metabolism, data-driven toxicology systems, and approaches enhancing interpretability and methodological reliability.

### Integration and alignment with new approach methodologies and regulatory policy

The FDA Modernization Act 2.0 (December 2022) formally authorizes non-animal alternatives (such as AI-based approaches and organ-on-chip technologies) to support Investigational New Drug submissions and marketing approval decisions, thereby codifying practices that had already begun to emerge in regulatory review. 90​ Recent FDA guidance materials also outline pathways for integrating and qualifying alternative approaches, including AI and microphysiological systems, for egulatory use. 91​ ​92​

Organs-on-chip and microphysiological systems enhance the physiological relevance of key organs, such as the liver and heart. These technologies are increasingly positioned as complementary new approach methodology (NAM) platforms that strengthen in-silico triage and improve confidence in high-impact end points such as DILI. 93​

Psychiatric drugs are disproportionately associated with metabolic adverse effects, QTc prolongation, and drug–drug interactions due to extensive CYP450 metabolism and frequent polypharmacy in this population. Early integrated ADMET modeling that accounts for these liabilities, especially BBB permeability, hERG channel inhibition, and CYP inhibition profiles, can help de-risk CNS candidates before costly late-stage failures.

## Artificial intelligence in clinical development and trial design

Clinical development remains the most extensive, costliest, and riskiest phase within the pharmaceutical R&D continuum. This section synthesizes how AI is enhancing patient identification and trial feasibility; optimizing trial design and analysis (including adaptive, covariate-adjusted, and digital-twin-assisted approaches) and improving data acquisition through digital health technologies (DHTs), all within evolving regulatory frameworks such as ICH E6(R3), relevant FDA guidance, and the EMA reflection paper. 10​ 94​​ ​95​

### Patient stratification and recruitment

Transitioning from manual prescreening to AI-assisted matching, natural language processing (NLP) and ML applied to EHRs and clinical registries can map complex inclusion and exclusion criteria to real-world patients, substantially expanding the pool of correctly identified candidates. In three oncology trials, AI-assisted prescreening using Mendel.ai increased the number of correctly identified potentially eligible patients by 24–50% compared with standard methods, with no patients being missed, illustrating a practical improvement during feasibility and prescreening. 96​

Beyond identification, computational frameworks such as Trial Pathfinder use real-world data to systematically relax or refine eligibility criteria and simulate their impact on outcomes (e.g., overall survival hazard ratios) and cohort sizes. In advanced non-small cell lung cancer, Pathfinder analyses demonstrated that relaxing non-causal exclusions can markedly expand eligibility while preserving comparable outcome distributions, suggesting that appropriately designed flexibility can accelerate accrual without compromising effect estimation. 97​

Electroencephalography (EEG)/functional magnetic resonance imaging (fMRI)-based stratification: ML models using baseline EEG and fMRI data are increasingly applied to predict antidepressant outcomes. In independent cohorts (CAN-BIND and EMBARC), EEG-based models have been externally validated with approximately 64% balanced accuracy for SSRI response. Similarly, the resting-state fMRI connectivity of the subgenual ACC predicts remission after escitalopram. Recent EMBARC-derived multimodal models integrating EEG, imaging, and immune markers demonstrate general prognostic value. Prospective, locked-model analyses embedded within randomized trials represent the next essential step. 98​ 99​ 100​ ​101​

Complementing neuroimaging approaches, speech and language analysis is emerging as a low-cost, scalable modality well suited to ML-based stratification. Automatically extracted acoustic and linguistic features, such as prosody, pause patterns, and semantic content, have been associated with neuropsychiatric symptom severity in MCI populations and with negative-symptom burden in schizophrenia, suggesting that voice-based measures may serve as transdiagnostic digital biomarkers for both enrichment and remote monitoring in psychiatric and neurodegenerative trials. 102​

### Adaptive design, covariate adjustment, and randomized controlled trial (RCT) augmentation

Adaptive designs are now well established under the FDA’s 2019 final guidance and the complex innovative design pilot guidance, which enables early interaction with the agency on model-informed proposals. The newly adopted ICH E6(R3) (Step 4, January 6, 2025) embeds risk-based, context-of-use (COU) principles for digitalized, model-informed trials. Further clarity was provided in June 2025 by the draft ICH E20 (Adaptive Designs) guidance, which elaborates on planning and operational principles. 10​ 94​​ 103​​ ​104​

Bayesian response-adaptive platforms (such as I-SPY 2 in high-risk breast cancer) demonstrate how adaptive randomization and biomarker-guided subgrouping can accelerate “go/no-go” decisions across multiple arms using pathological complete response as a surrogate end point. Several regimens have “graduated” to subsequent testing. Although not an AI method per se, such designs naturally accommodate AI-derived priors, predictive signals, and patient stratifiers. 105​ ​106​

The FDA’s 2023 guidance recommends prespecified adjustment for prognostic baseline covariates in randomized trials to improve precision in both superiority and non-inferiority settings, using linear or non-linear models. This framework allows the inclusion of locked prognostic scores as baseline covariates, provided such scores are prespecified before unblinding. 107​

The EMA issued a final Qualification Opinion (2022) for PROCOVA. This three-step methodology uses patient-specific prognostic scores to adjust for covariates in Phase 2/3 continuous-outcome trials. This established a regulatory-acceptable framework for increasing statistical power or reducing required control-arm size without an inflating Type I error. 108​

Extending the logic of prognostic covariate adjustment, AI-generated digital twins use ML models trained on historical trial and observational data to predict each participant’s counterfactual placebo trajectory. Including these individualized predictions as covariates can further reduce residual variance and reduce the number of control arms required. In a retrospective application to an Alzheimer’s disease Phase 2 trial, digital twins generated using a conditional restricted Boltzmann machine reduced the total residual variance by approximately 9–15% and enabled projected total sample size reductions of 9–15%, with control-arm reductions of 17–26%, while maintaining statistical power. 109​ Both the FDA and EMA have accepted digital twin-based methodologies for application in clinical trials. A recent perspective further outlines how digital twins, synthetic patient data, and in silico trial simulations can complement conventional designs, particularly in populations where recruitment is challenging. 110​ These approaches are especially promising for psychiatric trials, where high placebo response rates and end point variability inflate required sample sizes; however, their validity depends on the representativeness and quality of historical training data, and prospective validation in psychiatric populations remains essential.

### Digital health technologies and digital end points

Regulatory frameworks for remote data acquisition are now well established: the FDA’s 2023 DHT guidance details expectations for wearable and smartphone-based sensors covering validation, reliability, and data governance, 111​ and the EMA has qualified wearable-derived end points, most notably the stride velocity 95th centile (SV95C) as a primary end point in Duchenne muscular dystrophy, setting a precedent for sensor-derived clinical outcome assessments in drug development. 112​ 113​ ​114​

In psychiatry, individualized normative modeling is clarifying heterogeneity in psychosis. Person-level deviations in cortical thickness are sparse and idiosyncratic, arguing against case–control averages as decision-making tools and motivating the development of subject-specific biomarkers. Parallel studies demonstrate that speech-based indices (timing, pauses, and prosody) correlate with negative-symptom burden, providing scalable, remotely capturable measures for enrichment or secondary end points, pending multi-site validation and bias audits. 115​ 116​ 117​ ​118​

Ambulatory digital phenotyping has matured from feasibility to multi-site cohorts. In RADAR-MDD and related studies, smartphone and wearable features (sleep/circadian regularity, activity, and phone use) show convergent associations with depression severity and permit long-term remote follow-up, while systematic reviews have reported consistent links between circadian disruption and higher symptom burden, emphasizing the need for external validation and control for seasonality. 119​ 120​ 121​ ​122​

AI that transforms the raw sensor or imaging data into validated measures, such as computer vision-based scores or passive mobility metrics, can shorten readout times and enhance sensitivity, provided that these systems undergo fit-for-purpose validation and maintain human oversight in line with the EMA reflection paper. 95​

### Safety monitoring and post-market signal detection

AI methods now support pharmacovigilance and post-authorization safety by mining EHRs, insurance claims, spontaneous reports, and narrative texts. The FDA’s Sentinel Initiative exemplifies large-scale, data-driven safety surveillance. The FDA’s 2023 discussion paper (revised 2025) further outlines AI applications in trial simulation, dose optimization, and signal detection. 123​ ​124​ Recent reviews highlight maturing NLP and ML workflows for adverse-event detection and signal management, while emphasing transparency and rigorous evaluation as prerequisites for regulatory acceptance. 125​ ​126​

### Equity and bias in clinical development

Regulators increasingly expect proactive plans to improve representativeness in clinical development, such as diversity action plans. 127​

Amid evolving 2025 policies, sponsors are expected to document: (i) quantitative enrollment targets, (ii) site and network strategies to reach underrepresented populations, and (iii) bias audits for AI tools used in prescreening or outcome modeling, consistent with ICH E6(R3) and the EMA AI Reflection Paper, emphasizing human-centered, risk-based governance. 10​ 95​​ ​127​

## Industry landscape and translational case studies

AI-native (“AI-first”) biotechnology companies and technology-enabled pharmaceutical firms now represent a distinct and rapidly maturing tier within the global R&D ecosystem. Nevertheless, investments and development programs are still concentrated in oncology and neurology, predominantly in high-income regions, representing an equity gap that policymakers and funding agencies are only beginning to address through targeted initiatives. 128​ ​129​

### Sector size, geography, and business models

By early 2023, roughly 900 AI-focused biotechnology firms were active worldwide, underscoring rapid multi-year growth, although exact counts vary by how “AI-first” is defined. 130​ The United States hosts over half of these companies, followed by hubs in the UK and EU and a fast-growing presence in China and East Asia. 130​ Large pharmaceutical firms continue to favor multi-program discovery alliances over acquisitions: among the top 20 pharmaceutical companies, at least 55 AI partnerships were signed within 5 years, with>US $770 million upfront and US $38 billion in potential milestones. 131​ Table 2 presents representative examples of recent AI–biopharma collaboration agreements, with corresponding references provided in the table. 132​ 133​ 134​ ​135​

**Table:** **Table 2** Representative AI–biopharma and compute–biotech collaboration deals (2021–2025)

Announced	Parties	Deal type/scope	Headline economics	Notes (why notable)	Ref.
Dec 2021	Roche – Recursion	Decade-long collaboration in neuroscience and oncology	Up to “several billion” (exact figure n/d)	Often cited flagship “techbio×big pharma” example	132​
Jan 2022	Sanofi–Exscientia	Multi-program discovery partnership in oncology and immunology	Up to US$5.2B (headline value)	Illustrates the large-pharma preference for multi-program alliances	133​
Jul 2023	NVIDIA – Recursion	Strategic investment and co-development of foundation models for chemistry/biology	US$50M equity investment	Shows convergence of compute and biotech	134​
Jan 2024	Isomorphic Labs (Alphabet) – Eli Lilly and Novartis	Dual collaborations (milestone-based)	Nearly US$3B total (milestone-structured)	Alphabet’s entry into major pharma partnerships	135​

Note: Representative examples of major AI–pharma collaborations illustrating the sector’s shift toward multi-program discovery alliances and compute–biotech convergence.

### Artificial intelligence-designed molecules in psychiatric drug development

DSP-1181, a long-acting 5-HT1A agonist for obsessive-compulsive disorder, represents the first AI-designed small molecule to enter human studies in the CNS domain. Exploratory research was completed in less than 12 months, with a lead compound identified after approximately 350 syntheses compared with the several thousands typically required; Phase I was initiated in 2020. 47​ The collaboration subsequently advanced DSP-0038, a dual 5-HT2A antagonist and 5-HT1A agonist into clinical testing for neuropsychiatric symptoms. 136​ These programs illustrate how AI-guided iterative design can compress timelines and reduce synthesis burden, specifically in psychiatric drug development, where multi-target selectivity across aminergic receptors is a key design challenge.

### Cross-therapeutic context

Beyond psychiatry, AI-enabled pipelines have demonstrated translational progress in other domains. Insilico Medicine’s rentosertib (INS018_055), a TNIK inhibitor for idiopathic pulmonary fibrosis, was nominated approximately 18 months after AI-based target identification and reached first-in-human studies within 30 months at a reported cost of approximately US $2.6 million; a randomized Phase IIa trial published in 2025 demonstrated safety, tolerability, and dose-dependent efficacy signals, representing the first peer-reviewed proof-of-concept for a fully AI-discovered target–molecule pair. 137​ 138​ 139​ ​140​ Separately, Benevolent AI’s KG identified baricitinib as a potential COVID-19 therapy within 48 hours in January 2020, and it later received FDA Emergency Use Authorization that year, providing a clear example of AI-accelerated drug repurposing under pandemic timelines. 32​ 33​ 34​ 35​ ​36​ While neither example is psychiatric, both illustrate generalizable principles, including accelerated target to clinic translation and rapid hypothesis generation from KGs, that are directly applicable to CNS drug development.

Independent trackers indicate that dozens of AI-enabled new molecular entities have entered human testing, with an estimated Phase I completion rate of approximately 80–90% among reported programs. 83​ ​141​ However, these figures should be interpreted with caution, given variable definitions of “AI-designed” molecules, likely survivorship and publication bias, and the absence of controlled comparisons with non-AI programs.

### Therapeutic focus and equity implications

AI-related R&D investment remains concentrated in oncology and neurology, with a temporary surge in COVID-19 projects during 2020–2022. A global mapping analysis estimated that about 70% of AI R&D between 2018 and 2022 targeted these areas, reflecting strong market incentives and the relative abundance of high-quality datasets. 131​

Adoption of AI in drug discovery remains markedly higher in high-income countries (HICs) than in low- and middle-income countries (LMICs). A Wellcome Trust/BCG survey found regular AI use among 42% of HIC respondents, versus about 19% in LMICs, a disparity consistent across studies. Funding flows are similarly concentrated in HICs and China, limiting the near-term application of AI-enabled discovery to diseases primarily affecting LMIC populations. 131​

Global health authorities now emphasize trustworthy, risk-proportionate deployment of AI. The WHO’s 2024 guidance on large multimodal models highlights equity, transparency, and lifecycle oversight as core principles for AI in biomedical research and clinical development, standards that align closely with emerging expectations for AI-enabled discovery and trial technologies. 142​

Within psychiatry, the equity gap is especially pronounced: clinical trial populations for antidepressants and antipsychotics have historically underrepresented racial and ethnic minorities, and global access to novel AI-designed CNS therapeutics remains limited. Ensuring diverse representation in both training datasets and clinical validation cohorts is essential for generalizability of AI-enabled psychiatric drug development.

## Policy, ethical considerations, and future outlook

Global regulators are converging on risk-based, COU-specific expectations for AI systems in drug development. Table 3 summarizes key initiatives from ICH, the FDA, EMA, PMDA, WHO, and the EU. 10​ 95​​ 142​​ 143​ 144​ 145​ 146​ 147​ 148​ 149​ 150​ ​151​

**Table:** **Table 3** Global regulatory initiatives on AI transparency and governance (as of 2025)

Agency/region	Document/framework	Date/status	Scope and focus	Key principles and messages	Reference
ICH	E6(R3) GCP	Step 4 (adopted), Jan 6, 2025	Applicable to clinical trials and model-informed approaches	Embeds lifecycle quality management, proportionate oversight, and digitalization-ready principles including prespecification, documentation, and change control	10​
FDA	Draft Guidance: Considerations for the Use of Artificial Intelligence to Support Regulatory Decision-Making for Drug and Biological Products	Draft, Jan 2025	AI systems supporting regulatory decision-making	Proposes a risk-based credibility framework aligned to COU, covering data provenance, model development, verification/validation, uncertainty management, and lifecycle monitoring	144​
EMA	Reflection Paper on AI	Finalized, Sept 9, 2024	Medicines lifecycle from development to post-authorization	Highlights human oversight, data governance, and bias mitigation; cautions that large language models may generate plausible yet erroneous content, requiring close supervision in documentation workflows	95​
PMDA / MHLW (Japan)	Digital data practices and related guidance (e.g., ADaM, Define-XML, MID-NET, registry reliability, external controls, and e-consent)	Updated 2023–2024	e-Submissions, real-world data, decentralized elements	April 2024 updates aligned conformance standards for e-submissions; October 2024 “Points to Consider” clarified registry reliability and external control use; 2023 guidance enabled e-consent; December 2023 update allowed inclusion in MRCTs without domestic Phase 1 when justified by foreign safety data. Japan’s stance remains risk- and evidence-based, promoting digital submissions and real-world evidence with robust documentation and early regulatory consultation	143​ 145​​ 146​ 147​ 148​ 149​ ​150​
WHO	Ethics and Governance of Large Multimodal Models (LMMs) in Health	2025	Health research and clinical applications of foundation models	Emphasizes equity, transparency, accountability, and lifecycle risk management for foundation models in health	142​
European Union	EU AI Act	Took effect Aug 1, 2024	General-purpose and high-risk AI systems	Establishes phased implementation: prohibitions within 6 mo, obligations for general-purpose AI in 12 mo, high-risk compliance in 24 mo, and sector-specific requirements in 36 mo. Developers applying AI in medical contexts should map each use case to the appropriate risk regime and prepare for conformity assessment and post-market monitoring	151​

Abbreviations: AI, artificial intelligence; COU, context-of-use; eIC, electronic informed consent; EMA, European Medicines Agency; FDA, U.S. Food and Drug Administration; GCP, good clinical practice; ICH, International Council for Harmonisation; LMM, large multimodal model; MHLW, Ministry of Health, Labour and Welfare; MRCT, multi-regional clinical trial; PMDA, Pharmaceuticals and Medical Devices Agency; RWD/E, real-world data/evidence; V&V, verification and validation; WHO, World Health Organization.

Note: Major regulatory and policy developments shaping the AI use in drug development, emphasizing COU-specific expectations, transparency, traceability, and human accountability.

Translating these frameworks into practice requires addressing several intertwined challenges: non-representative training data (particularly Eurocentric genetic datasets) that risk widening health disparities; 152​ 153​ ​154​ opacity of complex models that hinders regulatory scrutiny despite advances in explainable AI; 88​ ​89​ persistent gaps in data interoperability and lifecycle monitoring; 155​ ​156​ and the tendency of generative chemistry systems to produce physically implausible outputs without physics-based filtering. 63​

Table 4 operationalizes these regulatory and ethical principles into a practical, stage-keyed governance checklist for psychiatric drug R&D, distinguishing established expectations from aspirational best practices.

**Table:** **Table 4** AI governance and implementation checklist for psychiatric drug R&D

R&D stage	Psychiatric challenge	Established expectation	Aspirational best practice
Target identification	Polygenic architecture; incomplete causal biology	Cross-validated genetic evidence (report PP3/PP4); prespecified negative controls and leakage audits for KG-based hypotheses 28​ ​29​	Multi-omics KGs with degree-confounding adjustment and cross-modal triangulation 39​ ​40​
Molecular design	BBB penetration; off-target aminergic/cholinergic activity; polypharmacy DDI risk	Synthetic feasibility checks for generative outputs; OECD QSAR principles 55​ 56​ 57​ 58​ 59​ 60​ 61​ ​62​	Multi-objective models with BBB, hERG, and CYP constraints; PoseBusters-style plausibility validation 63​
Preclinical safety	Metabolic adverse effects; QTc liability; CYP-mediated DDIs	Applicability-domain reporting; data provenance documentation per NIST AI RMF 159​	Integration with NAM platforms (cardiac organoids, liver-on-chip) for mechanistic confirmation 93​
Patient stratification	Phenotypic heterogeneity; diagnostic overlap; comorbidity	Prespecified, locked prognostic models validated on independent cohorts 98​ 99​ 100​ ​101​	Multimodal stratification (EEG, fMRI, speech/voice biomarkers) with subgroup performance reporting
Trial design	High placebo response; subjective end points; site variability	Prespecified analysis plans; regulatory alignment with FDA adaptive design guidance, ICH E6(R3), ICH E20 10​ 94​​ 103​​ ​104​	Digital twin augmentation with prospective validation in psychiatric populations; PROCOVA-style covariate adjustment 108​
Digital end points	End point noise; limited objective measures	Fit-for-purpose validation per FDA DHT guidance; 111​ human oversight per EMA reflection paper 95​	Regulatory qualification as primary end points; speech-based and passive digital COAs with multi-site validation 102​
Post-market safety	Polypharmacy interactions; long-term metabolic effects	Transparent pharmacovigilance workflows with human-in-the-loop review 95​ ​142​	Automated NLP-based signal detection integrated with lifecycle model monitoring 125​ ​126​
Governance /equity	Eurocentric datasets; underrepresentation in psychiatric trials	COU-based credibility plans; diversity action plans with enrollment targets 127​	Federated learning for cross-institutional training; subgroup-specific bias audits; equity-by-design across data collection and site selection 160​ ​161​

Abbreviations: AI, artificial intelligence; BBB, blood–brain barrier; COA, clinical outcome assessment; COU, context-of-use; CYP, cytochrome P450; DDI, drug–drug interaction; DHT, digital health technology; EEG, electroencephalography; FDA, U.S. Food and Drug Administration; fMRI, functional magnetic resonance imaging; hERG, human ether-à-go-go-related gene; ICH, International Council for Harmonisation; KG, knowledge graph; NAM, new approach methodology; NIST AI RMF, National Institute of Standards and Technology AI Risk Management Framework; NLP, natural language processing; OECD, Organisation for Economic Co-operation and Development; PP3/PP4, posterior probability of shared/distinct causal variants; PROCOVA, prognostic covariate adjustment; QSAR, quantitative structure–activity relationship; R&D, research and development.

Note: Practical checklist mapping key stages of psychiatric drug R&D to their domain-specific challenges, established regulatory expectations, and aspirational best practices. Established expectations reflect current regulatory guidance or widely accepted standards; aspirational practices represent emerging approaches supported by evidence but not yet formally required.

Achieving globally responsible AI further requires expanding non-European datasets with published portability metrics, 152​ ​154​ building technical capacity and privacy-preserving collaboration frameworks in LMICs consistent with WHO guidance, 142​ and ensuring that regulatory implementation, such as the EU AI Act, does not disproportionately burden smaller or resource-limited institutions. 151​

## Conclusions

AI has evolved from proof-of-concept to an end-to-end enabler that enhances decision quality across drug development. From target discovery to clinical validation, AI strengthens each stage, prioritizing genetically supported biology, generating chemically and physically plausible compounds, identifying ADMET risks through explainable models, and optimizing clinical trials via adaptive designs, covariate adjustment, and validated digital end points.

Looking ahead, three developments will shape the next phase. First, large multimodal foundation models are expanding across literature, multi-omics, and imaging workflows, while structure-aware systems such as AlphaFold 3 extend interaction modeling to complexes and ligands; successful deployment will require prespecified evaluation plans and independent external validation. 21​ Second, following the FDA Modernization Act 2.0, regulators are formalizing roadmaps to integrate NAMs, including organ-on-a-chip and in silico approaches, into non-clinical safety assessments, with AI increasingly triaging and contextualizing NAM outputs. 90​ ​157​ Third, FDA centers have begun deploying AI internally to assist reviewers, signaling institutional readiness for AI-augmented evaluation workflows. 158​

Regulators now define risk-based, COU frameworks that enable integration of model-informed evidence without sacrificing rigor. To translate these principles into actionable guidance for psychiatric drug development, where challenges such as phenotypic heterogeneity, high placebo response, subjective end points, and polypharmacy complicate every stage, Table 4 provides a stage-by-stage implementation checklist. Two imperatives guide this transition: reducing drug loss by improving candidate quality through causal, structure-aware, and NAM-aligned design, and shortening drug lag via AI-assisted trial execution. Early data suggest higher first-in-human success and shorter cycles, although broad external validation remains essential.

Equity must be intentional: expanding non-European datasets, enabling privacy-preserving collaboration with LMIC partners, and mandating transparency and subgroup auditing are vital to prevent data-rich bias.

Collectively, these advances position AI not merely as a productivity tool but as a catalyst for higher-quality, more equitable, and trustworthy drug discovery and development.
